# Test-retest reliability of the eating disorder examination-questionnaire (EDE-Q) in a college sample

**DOI:** 10.1186/2050-2974-1-42

**Published:** 2013-11-20

**Authors:** Jennifer S Rose, Adin Vaewsorn, Francine Rosselli-Navarra, G Terence Wilson, Ruth Striegel Weissman

**Affiliations:** 1 Department of Psychology, Wesleyan University, 207 High Street, Middletown, CT 06459, USA; 2 Department of Psychology, Manchester Community College, Great Path MS#4, PO Box 1046, Manchester, CT 06040, USA; 3 Graduate School of Applied and Professional Psychology, Rutgers – the State University of New Jersey, 152 Frelinghuysen Road, Piscataway, NJ 08854-8020, USA

**Keywords:** Eating disorders, EDE-Q, Test-retest reliability, Undergraduate men and women

## Abstract

**Background:**

The Eating Disorder Examination-Questionnaire (EDE-Q), a widely used self-report instrument, is often used for measuring change in eating disorder symptoms over the course of treatment. However, limited data exist about test-retest reliability, particularly for men. The current study evaluated EDE-Q 7-day test-retest reliability in male (n = 47) and female (n = 44) undergraduate students together and separately by gender.

**Results:**

Internal consistency was consistently higher for women and at Time 2, but remained acceptable for both men and women at both time points. Cronbach’s α ranged from .75 (Restraint at Time 1) to .93 (Shape Concern at Time 2) for women and from .73 (Eating Concern at Time 2) to .89 (Shape Concern at Time 2) for men. With the exception of some of the eating disorder behaviors, test re-test reliability was fairly strong for both men and women. Shape Concern and the global EDE-Q score were highest for both men and women (Spearman’s rho > 0.89 with the exception of Shape Concern for women for which Spearman’s rho = .86). Test re-test reliability was lower for the eating disorder behavior measures, particularly for men, for whom Kendall’s tau-b for frequency and phi for occurrence was less than 0.70 for all but objective bulimic episodes.

**Conclusions:**

Results were consistent with past research for women, indicating strong test re-test reliability in attitudinal features of eating disorders, but lower test re-test reliability in behavioral features. Internal consistency and test re-test reliability was good for the attitudinal features of eating disorder in men, but tended to be lower for men compared to women. The EDE-Q appears to be a reliable instrument for assessing eating disorder attitudes in both male and female undergraduate students, but is less reliable for assessing ED behaviors, particularly in men.

## Background

The EDE-Q [1] is a widely used measure to assess eating disorder (ED) attitudes and behaviors in both community and clinical populations. Eating disorders are especially prevalent among college women and are becoming more prevalent among young men [2]. Consequently, identifying students with eating disorders is important so that treatment can be made available to these students. The EDE-Q is a particularly useful measure to assess eating disorder attitudes and behavior in the broader population of college students as it is easy and inexpensive to administer and can quickly measure eating disorders and compensatory behaviors in large samples. However, since assessment for detection of eating disorders in college students is likely to occur infrequently in non-research university settings, temporal stability is a critical component of any ED measure used for this purpose.

In a recent literature review on the psychometric properties of the EDE-Q, Berg, Peterson, and colleagues noted that there were relatively few studies that examined the reliability of the EDE-Q [3]. Table 1 provides information on EDE-Q test re-test studies based on a review of the literature for the current study. Even fewer studies have examined test-retest reliability in US college students and these studies evaluated EDE-Q reliability for women only (Table 1). Although norms have been developed for college men [4], there are no published studies specifically examining EDE-Q test re-test reliability for this population. Finally, to our knowledge, there are no published studies that evaluate test-retest reliability of frequency and occurrence of ED behavioral features in men. The purpose of this study is to evaluate test-retest reliability of the EDE-Q in a nonclinical population of male and female college students as a whole, as well as separately by gender.

**Table 1:** Studies assessing EDE-Q test-retest reliability

**Authors/study year**	**Study title**	**Sample**	**Test re-test time frame**	**Reliability statistics**	**Results**
Luce & Crowther [5]	The Reliability of the Eating Disorder Examination--Self-Report Questionnaire Version	N = 139 female undergraduates at a large midwestern university	14 days	Phi coefficient (items measuring key behavioral features of eating disorders)	Reliability of EDE-Q items measuring occurrence and frequency of behavioral features:
		18.5 years old on average (SD = 2.0)			
					-Occurrence (Phi coefficient) = Binge eating .62 Self-induced vomiting .66
		86% white, 8.4% African American, 2.0% Hispanic, 1.0% Native American, 2% other			
				Pearson r (test re-test reliability of items measuring frequency of behavioral features and EDE-Q subscales)	Laxative misuse .70 Diuretic misuse .57
		97% single, 2% married, 1% separated or divorced Avg. BMI = 22.5 (SD = 4.0)			-Frequency (Pearson r) = Binge eating .68 Self-induced vomiting .92
		Recruited through offering extra credit points toward research assignment in Introductory Psychology course; one additional extra credit point offered to participants willing to return for a second session (68% did)			
					Laxative misuse .65 Diuretic misuse = .54 -Cronbach’s alpha =
				Cronbach’s alpha (internal consistency of subscales)	Restraint T1 .84/T2 .85 Shape Concern T1 .93/T2 .92 Weight concern T1 .89/T2 .89
					Eating concern T1 .78/T2 .81- test-retest reliability of EDE-Q subscales Pearson r: Restraint .81 Shape concern .94 Weight concern .92 Eating concern .87
Mond, Hay et. al. [6]	Temporal Stability of the Eating Disorder Examination Questionnaire	802 women aged 18-45. Recruited in two phases: (1) selected at random from the national (Australia) electoral roll and sent an EDE-Q, self-report weight and height questionnaire and demographic form (2) Of those participants, all who completed the questionnaires, provided a phone number and indicated a willingness to be contacted by telephone at a later date were selected to participate in the second administration of the EDE-Q	315 days	Kendall’s tau-b (frequency)	-Range of Cronbach’s alpha coefficients for individual subscales:
				Phi coefficient (occurrence)	
					-Eating Concern = .73 to Shape Concern = .87
				Cronbach’s alpha (internal consistency)	
					Global score = .93
					-Eating disorder behaviors occurrence/frequency test re-test correlations (Phi Coefficient and Kendall’s’ tau-b):
					-Objective bulimic episodes: Occurrence (phi) .44 and Frequency (Kt-b) .44
					-Subjective bulimic episodes = Occurrence .24 and Frequency .28-Exercising for shape or weight = Occurrence .31 and Frequency .31
Reas,Grilo, & Masheb [7]	Reliability of the Eating Disorder Examination-Questionnaire in patients with binge eating disorder	N = 86 men and womenAvg. age = 23–59 (mean = 44.9, SD = 8.9)	Mean = 4.8 days Range = 1–14 days	Spearman’s rho (test retest reliability)	Overeating behaviors
					-OBEs = .84
					-SBEs = .51
		79.1% female, 20.9% male			-OOEs = .39
		82.6% Caucasian			Subscales
					-Restraint = .77
					-Shape concern = .66
		66% married			-Weight concern = 71
		51.8% college graduates Mean BMI = 36.9			-Eating concern = .72
					EDE-Q total score = .76
					Subscales at different time lag intervals
		Participants recruited through print advertisements for treatment studies of BE at a university med school; pre-screening criteria included age 18–60, BMI > 27, likely BED diagnosis; exclusionary criteria included concurrent eating/weight/psychiatric treatment, medical conditions that influence weight			
					Overeating Behaviors:-OBEs =
					.82 (0–1 days), .86 (2–14 days), .82 (7–14 days)
					-SBEs =
					.58 (0–1), .41 (2–14), .37 (7–14)
					-OOEs =
					.51 (0–1), .34 (2–14), .19 (7–14)
					-Restraint =
					.79 (0–1), .86 (2–14), .82 (7–14)
					-Shape concern =
					.79 (0–1), .75 (2–14), .66 (7–14)-Weight concern =
					.76 (0–1), .70 (2–14), .71 (7–14)
					-Eating concern =
					.69 (0–1), .72 (2–14), .77 (7–14)
					EDE-Q total score =
					.79 (0–1), .74 (2–14), .72 (7–14)
Elder & Grilo [8]	The Spanish language version of the Eating Disorder Examination Questionnaire: Comparison with the Spanish language version of the eating disorder examination and test-retest reliability	N = 77 Latina women (monolingual Spanish-speakers) recruited through print advertisements	Mean = 8.9 days (SD = 2.5, range = 5–14 days)	Spearman’s rho (test re-test reliability)	Subscales
					-Restraint: Spearman rho = .59
					-Eating concern: .81
		Avg. age 41.5 (sd = 13.6 -Mean BMI = 29.1 (sd = 5.9; range 19.8-43.0)			-Weight concern: .71
					-Shape concern: .81
					-Global score: .85
Bardone-Cone & Boyd [9]	Psychometric Properties of the Eating Disorder instruments in Black and White young women: Internal consistency, temporal stability, and validity	N = 97 Black and N = 179 White female undergraduates.	Mean = 5.24 months	Cronbach’s alpha	Cronbach’s alpha range: .81 (Restraint) to .89 (Shape Concern) for Black women and .84 (Restraint and Weight Concern) to .91 (Shape Concern) for White women
				Pearson r (test retest reliability)	
		Oversampled for Black women. Recruited through introductory psychology classes and campus wide e-mail, flyers. Mean age black women = 19.0 (sd = 1.59); White women 18.6 (sd = 1.06)			
				Phi coefficient (occurrence)	
					Test-retest reliability
					Black women:
					Restraint = .57; Eating Concern = .79; Weight Concern = .81; Shape Concern = .82;
		N = 70 Black women and N = 156 White women with data at Time 2			
					OBE = .57; SBE = .19; Exercise = .31
					Test-retest reliability
					White women:
					Restraint = .71; Eating Concern = .81; Weight Concern = .81; Shape Concern = .80 OBE = .53; SBE = .40; Exercise = .39
Becker et al. [10]	Validity and Reliability of a Fijian Translation and Adaptation of the Eating Disorder Examination Questionnaire	N = 523 school-going adolescent Fijian females	Approximately 1 week	Intraclass correlation coefficient (subscales)	-ICC (English) = .79 (global); .75 (restraint) .55; (eating concern), .70 (shape concern), .78 (weight concern) -ICC (Fijian) .70 (global), .60 (restraint), .50 (eatingconcern), .63 (shape concern), .56 (weight concern
		N = 81 subjects who re-took the EDE-Q within ~1 wk; 21 retook EDE-Q in English, 60 in Fijian			
		Ages 15–20 from 12 secondary schools registered in one administrative sector in the Fiji Ministry of Education as of October 2006			
					-Kappa (English) .81 (any purging), .39 (vomiting), .48
					(laxative misuse), .51 (herbal purgative use), .53 (driven exercise), .68 (fasting), .55 (binge eating)
					-Kappa (Fijian) = .62 (any purging), .66 (vomiting), .13
					(laxative misuse), .63 (herbal purgative use), .46 (driven exercise), .61 (fasting), .60 (binge eating)
				Kappa (behaviors)	
Ro, Reas, & Lask [11]	Norms for the Eating Disorder Examination Questionnaire among female university students in Norway	N = 671 women	-Mean = 8.3 days -SD = 2.8 days	Spearman’s rho (test retest reliability)	-Spearman rho = .93 (global EDE-Q score)
		Ages 18–66 (mean = 24.8, SD = 6.9)			
					.90 (restraint)
					.82 (eating concern)
					.91 (shape concern)
					.86 (weight concern)
					.83 OBEs
					.73 (self-induced vomiting)
					.81 (laxative misuse)
					.71 (excessive exercise)
		Self-reported avg. BMI was 22.3, SD =3.4 (range = 11.9-45.0)			
				Cronbach’s alpha (internal consistency)	
		61% unmarried and 29% cohabiting or unmarried			
		10.1% of students had immigrated to Norway and 37% originally from country outside of Europe			
		Recruited from five different departments in two university settings in Norway; given lottery ticket as compensation			
					-Cronbach’s alpha = .94 (global)
					.75 (restraint)
					.78 (eating concern)
					.90 (shape concern)
					.81 (weight concern)
Yucel et al. [12]	The Turkish version of the Eating Disorder Examination Questionnaire: Reliability and validity in adolescents	N = 925 primary and high school students 626 girls and 299 boys	-15 days or less	(test retest reliability)	-Pearson r = .91 (global score)
				Cronbach’s alpha (internal consistency)	
		Mean age = 15.52 years (SD = 1.88, range = 12-18)			.43 (binge eating)
			(mean not specified)		.89 (weight concern
		Test retest reliability carried out on 52 girls and 26 boys			
					.79 (restraint)
					.83 (eating concern)
					.89 (shape concern)
Pliatskidou et al. [13]	Reliability of the Greek version of the eating disorder examination questionnaire (EDE-Q) in a sample of adolescent students	N = 257 secondary school students 133 girls, 124 boys	Mean = 34 days	-Intraclass and Pearson r	-Cronbach’s alpha range .71 - .91
				(test-retest reliability for subscales and global score)	-Intraclass correlation coefficients = range .55 - .70
		Avg age = 16.1 (sd = 1.4)			-Pearson r range .58 - .73
				-Kendall’s tau-b (behavioral features)	-Kendall’s tau-b range .22 - .57

## Methods

### Participants

The EDE-Q was administered to N = 91 male (N = 47) and female (N = 44) undergraduate students recruited for research participation credit in a large introductory psychology course at a university in the northeastern United States. The mean age was 19 (sd = 1.16; range = 18-23). Participants were able to identify with more than one ethnicity. The majority (59%) of participants identified as White, 33% identified as Asian, 11% identified as Black, and 8% identified as Hispanic. For both assessments, students completed a paper and pencil self-report EDE-Q questionnaire in person during individually scheduled appointments. The average test re-test interval was 6.88 days (sd = 1.36 days, range = 5-14 days), and the test re-test interval was between 6 and 8 days for 94.5% of the participants. No other questionnaires besides the EDE-Q were completed at either time point. All but 3 participants completed both assessments. The study was approved by the university’s institutional review board.

EDE-Q V6.0 procedures (Fairburn, 2008) were used to score the EDE-Q at both time points. Subscale scores were created by averaging the corresponding items, provided that participants responded to more than half of those items. Subscales included Restraint (5 items), Eating Concern (5 items), Shape Concern (8 items), and Weight Concern (5 items). A global EDE-Q score was created averaging the 4 subscales. Both frequency (number of times) and occurrence (a binary variable representing engaging in the behavior at least one time; yes/no) of ED behavioral features (objective bulimic episodes (OBE), OBE days, objective overeating (OO) episodes, and exercise to control weight or shape) were examined, as was a composite behavior score which was an average of OBE, OBE days, and OO episodes frequency variables. Subjective binge eating episodes (SBE), which could be determined in earlier versions of the EDE-Q, cannot be determined in Version 6.0 of the EDE-Q. An SBE is defined as an occasion when there is a perceived loss of control, but the amount of food eaten is not large. The EDE-Q 6.0 assesses loss of control, but only with regard to occasions when a large amount of food is consumed. Because vomiting (N = 2) and laxative use (N = 1) were rare, test re-test reliability statistics were not computed for these variables.

### Analysis

Internal consistency was calculated using Cronbach’s coefficient alpha (α) for the four continuous EDE-Q subscales (Restraint, Eating Concern, Shape Concern and Weight Concern) and the global EDE-Q score. To facilitate comparison to previous studies, 7-day test-retest reliability of each continuous subscale, the global EDE-Q score, frequency of OO, OBE, and OBE days, and the binge behaviors composite score was estimated using Pearson r and Spearman’s rho statistics. It has been suggested that test retest reliability coefficients of .80 or higher for these statistics are indicative of acceptable test re-test reliability [14].

Kendall’s tau-b was also calculated for the ED behavior frequency variables due to more extreme nonnormality in these measures compared to the global EDE-Q score and the four subscales. In cases of extreme nonnormality, Kendall’s tau-b has been found to be superior to Spearman’s rho [15]. Kendall’s tau-b is a nonparametric test of rank association. Similar to the Pearson correlation coefficient and Spearman’s rho, Kendall’s tau-b can range from -1 (perfect disagreement) to +1 (perfect agreement). Although there is no well established criterion for acceptable test retest reliability for Kendall’s tau-b, its magnitude is generally lower by a ratio of Spearman’s rho to Kendall’s tau-b of approximately 3/2 due to differences in computation [16]. Finally, phi coefficients were calculated for the binary binge behavior occurrence variables. All statistics were calculated for the entire sample, as well as separately by gender.

## Results

### Descriptive statistics

Table 2 show means and standard deviations for the continuous measures, and the number and percentage of students indicating having engaged in the behavior at least once are shown for binge behavior occurrence. Means for women on the global EDE-Q score, Shape Concern, and Weight Concern were consistent with established EDE-Q norms for college women, but women in this study had slightly lower means than the norm on Restraint and Eating Concern [17]. The rate of reported OBE and Excessive Exercise was higher for women compared to the norm for college women, but lower for Vomiting and Laxative Use [17]. Men had slightly lower means on Eating Concern, Shape Concern, and Weight Concern compared to the norm for college men, but were consistent with the norm for Restraint [4]. Similar to women, the rate of Excessive Exercise was higher for men compared to the norm, but lower for Vomiting and Laxative Use, whereas the rate of reported OBE episodes for men was consistent with the norm [4].

**Table 2:** EDE-Q means (standard deviation) for continuous measures and percentages (N) for binary ED behavior occurrence at time 1 and time 2

	**Full sample (N = 91)**		**Men (N = 47)**		**Women (N = 44)**	
*Measure*	*Time 1*	*Time 2*	*Time 1*	*Time 2*	*Time 1*	*Time 2*
Restraint	1.24 (1.14)	1.08 (1.17)	1.07 (1.09)	0.87 (1.14)	1.41 (1.16)	1.30 (1.18)
Eating Concern^a^	0.65 (0.95)	0.63 (0.96)	0.37 (0.68)	0.37 (0.60)	0.94 (1.11)	0.90 (1.18)
Shape Concern^a^	1.80 (1.36)	1.69 (1.44)	1.35 (1.24)	1.30 (1.29)	2.27 (1.33)	2.09 (1.49)
Weight Concern^a^	1.39 (1.35)	1.33 (1.45)	0.97 (1.07)	0.93 (1.21)	1.84 (1.48)	1.75 (1.86)
Global EDE-Q^a^	1.27 (1.05)	1.18 (1.12)	0.95 (0.85)	0.87 (0.92)	1.62 (1.14)	1.51 (1.22)
*ED Behavior Frequency*						
OBEs	1.71 (4.14)	2.05 (6.29)	1.07 (2.76)	0.90 (1.80)	2.41 (5.19)	3.24 (8.70)
OBE days^b^	2.02 (4.28)	2.41 (4.41)	1.11 (2.75)	2.08 (3.34)	2.98 (5.31)	2.76 (5.33)
OO episodes^a^	4.31 (7.41)	3.82 (6.98)	5.83 (9.10)	5.93 (9.12)	2.71 (4.66)	1.77 (2.71)
Vomiting	0.42 (3.12)	0.44 (3.19)	0.00 (0.00)	0.00 (0.00)	0.86 (4.45)	0.91 (4.55)
Laxative use	0.00 (0.00)	0.01 (0.11)	0.00 (0.00)	0.02 (0.15)	0.00 (0.00)	0.00 (0.00)
Excessive exercise	5.02 (8.32)	2.97 (5.94)	5.60 (9.37)	2.78 (6.15)	4.43 (7.15)	3.15 (5.78)
ED Behaviors composite score	2.61 (3.34)	2.72 (4.09)	2.74 (3.42)	2.84 (3.30)	2.47 (3.30)	2.59 (4.82)
*ED Behavior Occurrence*						
OBEs	28.6% (26)	37.4% (34)	21.3% (10)	29.8% (14)	36.4% (16)	45.5% (20)
OBE days^b^	35.2% (32)	47.3% (43)	23.4% (11)	42.6% (20)	47.7% (21)	53.5% (23)
OO episodes^c^	56.0% (51)	60.4% (55)	63.8% (30)	70.2% (33)	47.7% (21)	50.0% (22)
Vomiting	2.2% (2)	2.2% (2)	0% (0)	0% (0)	4.5% (2)	4.7% (2)
Laxative use	0% (0)	1.1% (1)	0% (0)	2.2% (1)	0% (0)	0% (0)
Excessive exercise^c^	45.1% (41)	35.2% (32)	40.0% (18)	25.0% (11)	52.3% (23)	48.8% (21)

^a^Significant gender differences at both time points. ^b^Significantly gender difference at Time 1 only.

^c^Significantly gender difference at Time 2 only.

Men scored significantly lower at both time points than women on all EDE-Q subscales and global EDE-Q, with the exception of Restraint. Men reported fewer OBEs (mean = 1.07 and 0.90 Times 1 and 2, respectively) and OBE days (mean = 1.11 and 2.08 Times 1 and 2, respectively) compared to women. However, these differences were statistically significant only for OBE days at Time 2. Conversely, men reported significantly more OO episodes (mean = 5.83 and 5.93 Times 1 and 2, respectively) compared to women (mean = 2.71 and 1.77 at Times 1 and 2, respectively). Men had higher scores on the binge behaviors composite score due to their higher rates of OO. Vomiting and laxative use were rare. None of the participants reported using laxatives at Time 1 and only one male participant reported laxative use at Time 2. Two women reported vomiting to control shape or weight at Time 1 and Time 2. None of the men reported vomiting to control shape or weight. However, 45% of participants in Time 1 and 35% in Time 2 reported exercising to control shape or weight. There were no significant gender differences in frequency of excessive exercise, although women reported a significantly higher level of excessive exercise occurrence at Time 2.

### Internal consistency

Table 3 shows Cronbach’s α internal consistency for the four EDE-Q subscales. Internal consistency was acceptable for all four subscales. Overall, internal consistency was lower at Time 1 than Time 2 and lowest for Restraint at Time 1, yet remained acceptable at both time points for both men and women. Internal consistency was consistently higher for women, with the exception of Restraint at Time 2 (α = .86 for men and .81 for women). Cronbach’s α ranged from .74 (Restraint) to .89 (Shape Concern) for men and from .75 (Restraint) to .93 (Shape Concern) for women.

**Table 3:** Cronbach’s coefficient alpha values for EDE-Q subscales at Time 1 and Time 2

	**Full sample (N = 91)**		**Men (N = 47)**		**Women (N = 44)**	
Subscale	*Time 1*	*Time 2*	*Time 1*	*Time 2*	*Time 1*	*Time 2*
Restraint	0.73	0.83	0.74	0.86	0.75	0.81
Eating Concern	0.79	0.86	0.73	0.77	0.79	0.89
Shape Concern	0.87	0.92	0.86	0.89	0.87	0.93
Weight Concern	0.82	0.87	0.77	0.82	0.83	0.89
Global EDE-Q	0.89	0.90	0.83	0.87	0.91	0.92

### Test re-test reliability

Tables 4 and 5 show the test re-test reliability coefficients for the EDE-Q measures. With the exception of some of the ED behaviors, test re-test reliability was fairly strong for both men and women. Shape Concern and the global EDE-Q score were highest for both men and women (Spearman’s rho =0.89 or greater with the exception of Shape Concern for women for which Spearman’s rho = .86). Test re-test reliability was lower for the ED behavior measures, particularly for men, for whom Kendall’s tau-b for frequency and phi for occurrence was less than 0.70 for all but OBE. Among women, Kendall’s tau-b was less than .70 for all but Excessive Exercise frequency, although test re-test reliability for ED Behavior occurrence was more reasonable.

**Table 4:** EDE-Q 7-day test re-test reliability for continuous EDE-Q measures

	**Full sample (N = 91)**			**Men (N = 47)**			**Women (N = 44)**		
	***Pearson r***	***Spearman’s rho***	***Kendall’s tau-b***	***Pearson r***	***Spearman’s rho***	***Kendall’ tau-b***	***Pearson r***	***Spearman’s rho***	***Kendall’s tau-b***
*Subscale*									
Restraint	0.81	0.79		0.83	0.76		0.78	0.81	
Eating Concern	0.84	0.80		0.80	0.68		0.83	0.83	
Shape Concern	0.91	0.91		0.94	0.93		0.87	0.86	
Weight Concern	0.90	0.75		0.88	0.85		0.90	0.91	
Global EDE-Q	0.92	0.92		0.92	0.89		0.90	0.90	
*ED Behavior Frequency*									
OBEs	0.88	0.80	0.72	0.79	0.80	0.75	0.92	0.79	0.69
OBE days	0.78	0.61	0.55	0.36	0.41	0.38	0.93	0.79	0.69
OO episodes	0.92	0.70	0.60	0.95	0.75	0.63	0.76	0.60	0.54
Excessive exercise	0.77	0.73	0.73	0.68	0.72	0.65	0.89	0.88	0.79
Binge Behaviors Composite	0.90	0.78	0.56	0.80	0.75	0.61	0.88	0.84	0.73

**Table 5:** EDE-Q 7-day test re-test reliability for binary ED behavior occurrence

	**Full sample (N = 91)**	**Men (N = 47)**	**Women (N = 44)**
*ED Behavior Occurrence*	*Phi*	*Phi*	*Phi*
OBEs	0.74	0.78	0.70
OBE Days	0.65	0.51	0.78
OO episodes	0.69	0.61	0.71
Excessive Exercise	0.75	0.67	0.82

## Discussion

The current study examined internal consistency and 7-day test re-retest reliability among college men and women. Consistent with past research, internal consistency was reasonable for all four subscales and higher for the global EDE-Q measure [6,11]. Internal consistency was lowest for the Restraint subscale. Internal consistency was slightly lower for men compared to women, but still acceptable. Interestingly, internal consistency was higher for both men and women for Time 2 compared to Time 1. Given the relatively short 7-day interval between assessments, this might reflect greater familiarity with the EDE-Q at Time 2, thus producing a higher correlation among the attitudinal items.

Test re-test reliability was generally high for the four attitudinal subscales and the global attitudinal EDE-Q score, but lower for ED behavior frequency and occurrence. This is consistent with past research indicating greater temporal stability in ED attitudes compared to ED behaviors [5,9-12]. Men had lower test re-test reliability for ED attitudes and behaviors compared to women. This might reflect that, for many men, eating attitudes and behaviors may be more likely to be driven by a desire for muscularity [18]. Consequently, men may have different ED concerns and behaviors unmeasured by the EDE-Q that may influence the reliability of the EDE-Q constructs in men. For example, rather than overeating or binge eating to be thinner, some men may engage in these behaviors to build larger bodies with more muscle mass. The higher rate of overeating without perceived loss of control in men may be due in part to a conscious decision to eat more in order to increase muscle building. Further, research has indicated that men experience fewer shape and weight concerns than women [19], and this is supported by the lower scores on ED attitudes for men. Men may engage in more intermittent dieting behaviors related to muscle building, which might impact temporal stability of eating behaviors. To our knowledge, this is the first study that examined temporal stability of the EDE-Q in men. However, this study could not assess the validity of the measure in men. Consequently, more research examining both reliability and validity of the EDE-Q in men is warranted in order to replicate and understand the findings in this study, and more clearly determine the extent to which the EDE-Q is a valid measure for men.

Despite lower test reliability for ED behaviors compared to ED attitudes in this study, temporal stability of ED behaviors was higher compared to previous studies. This may be due to the short interval between assessments, which results in an overlap in recall of these behaviors because participants are asked to recall their behavior over the past 28 days. Test re-test reliability for ED behaviors has been found to decrease as the interval between assessments increases [7], and is often unacceptably low for test re-test intervals that extend over several months [6]. Establishing good temporal stability for a short interval is important, as it can be considered an upper limit on the stability of the EDE-Q because attitudes and behaviors are less likely to change over such a short period of time. If short term test retest reliability is poor, then observed changes in EDE-Q scores resulting from true changes in attitudes and behaviors that might occur over a longer period of time will be confounded with unreliability in the measure.

There are some limitations to this study that should be noted. First, the sample was too small to examine laxative use and vomiting to control shape or weight. This problem has plagued most past research as well [6,8,9,11]. Only a few studies have examined temporal stability in laxative use and vomiting, which have shown low to moderate temporal stability for these behaviors [5,10,11]. However, most of these studies were conducted on populations from countries other than the United States, and tended to have considerably larger samples sizes. Second, the test re-test reliability coefficients were calculated based on the originally proposed four factor structure for the EDE-Q subscales [1]. Although other studies examining the factor structure of the EDE-Q subscales have found a varying range of factors [20,21], we chose to examine test re-test reliability of the four original subscales in order to be comparable to other studies examining the psychometric properties of the EDE-Q. We did not collect body mass index (BMI) data in this study. It is reasonable to assume that there would be little to no change in BMI within individuals from the first to the second assessment only 7 days later. Consequently, BMI is not likely to have influenced test re-test reliability in this study because it likely to have remained stable between assessments. However, a lack of BMI data makes it more difficult to compare overall EDE-Q attitude and behavior scores in this study to scores in other studies. Finally, the current study relied on self-reports of ED attitudes and behavior, so it is possible that observed gender differences may be a function of differences in retrospective or other recall bias.

## Conclusions

This study examined test re-test reliability of the EDE-Q in college women and men, and is the first study to report test re-test reliability in men specifically. Results were consistent with past research for women, indicating good stability in attitudinal features of ED and lower stability in behavioral features for a relatively short 7-day test re-test interval. Internal consistency and test re-test reliability was good for the attitudinal features in men, but tended to be lower compared to women, particularly for the behavioral features of ED. This suggests that men are less consistent in their ED behaviors, possibly due in part to having different goals for ED behaviors. However more research is necessary to determine whether this is a reliable finding and whether it extends to longer test re-test intervals. This study indicates that the EDE-Q is a reliable instrument for assessing eating disorder attitudes in both male and female undergraduate students, but is less reliable for assessing ED behaviors, particularly in men for whom only OBEs appeared to have acceptable test re-test reliability.

## Competing interests

The authors declare that they have no competing interests.

## Authors’ contributions

JSR supervised the design of analyses, conducted some of the analyses, and wrote and revised the majority of the manuscript. AV conducted the majority of the analyses, wrote the first draft of the Results section and contributed to subsequent drafts. FRN, GTW, and SW conceptualized the study, and contributed significantly to all drafts of the manuscript. All authors read and approved the final manuscript.
